# Social and economic driving forces of recent CO_2_ emissions in three major BRICS economies

**DOI:** 10.1038/s41598-024-58827-9

**Published:** 2024-04-05

**Authors:** Eleni Koilakou, Emmanouil Hatzigeorgiou, Kostas Bithas

**Affiliations:** 1 Department of Economic & Regional Development, Institute of Urban Environment & Human Resources, Panteion University, 29 Aristotelous Street, Kallithea, 17671 Athens, Greece; 2 Center for Systems Integration & Sustainability, Michigan State University, Manly Miles Building, 1405 South Harrison Road, East Lansing, MI 48823-5243 USA; 3 Energy Management Laboratory, Department of Environment, University of the Aegean, University Hill, 81100 Lesvos, Greece

**Keywords:** Environmental impact, Energy and society, Environmental economics

## Abstract

The study examines the driving factors of total energy-related and power-related (electricity-heat) CO_2_ emissions for China, India and Brazil, three BRICS countries with vital economic and demographic dynamics. The paper applies decoupling and decomposition analysis in order to investigate the influence of those factors that are prominent in the contemporary literature as well as factors reflecting important social and demographic dynamics which affect the ecological footprint of society. Household size and number of households are introduced into the relevant literature for the first time to reflect demographic factors with substantially different trends from population size, the predominant factor in the existing literature. This novelty together with the simultaneous application of decoupling and decomposition analysis adds importance to the findings of the study, which covers the period of 2000–2018. The results show that increasing income and population significantly enlarge the energy-related CO_2_ emissions. Household size, number of households and income effects are crucial in the increase of power-related CO_2_ emissions. The crucial factor for the decrease of energy-related CO_2_ emissions is the decreasing energy intensity, while for power-related CO_2_ emissions it is the emission factor effect reflecting the recent shift towards less carbon intensive energy types.

## Introduction

Global energy-related CO_2_ emissions rose by 6% in 2021 to 36.3 billion tons, their highest ever level, as the world economy rebounded strongly from the Covid-19 crisis, relying heavily on coal to power that growth. The increase in global CO_2_ emissions of over 2 billion tons was the largest in history in absolute annual terms^1^.

In 2018 the five BRICS countries (Brazil, Russia, India, China and South Africa), accounted for 42% of global greenhouse gas emissions. China was the world's first-ranked emitter with 28% of the global total, with the United States a distant second with 15%. In third and fourth places came India with 7% and Russia with 5%. South Africa stood 13th and Brazil 14th with about 1% each (BRICS Research Group). Regarding the electricity-related emissions, in 2018 BRICS accounted for 46.2% of global power-related CO_2_ emissions, with Brazil 0.5%, India 8.6% and China 35.4%^2^.

For China, India and Brazil, several studies have investigated the driving factors of CO_2_ emissions. Paul and Bhattacharya attempted to identify the factors that influenced the trends in energy-related CO_2_ emissions in India for the period 1980–1996. Their results showed that economic growth had the largest positive effect on CO_2_ emissions^3^. Attari and Attaria studied the energy status of Pakistan, India and China by applying Decomposition Analysis (DA) for the years 1971–2008. The analysis identified energy intensity as the most important driving factor contributing to the reduction of CO_2_ emissions for India and China, while income was the factor contributing most to the increase of CO_2_ emissions^4^.

Das and Paul applied DA to the changes in CO_2_ emissions between 1993–1994 and 2006–2007 in India to identify the driving factors of CO_2_ emissions in the household sector. The study indicated that economic activity, structure and population effects were the main causes of increased CO_2_ emissions^5^. Yeo et al. attempted to identify and analyze the key drivers behind changes of CO_2_ emissions in the residential sectors of China and India by applying the Logarithmic Mean Divisia Index (LMDI) technique from 1990 to 2011. Their results indicated that changes in the population and energy consumption drove the increase in CO_2_ emissions^6^.

Rüstemoglu and Andrés dealt with the DA of energy-related CO_2_ emissions in Brazil from 1992 to 2011. The major factor associated with the increase of CO_2_ emissions in Brazil’s economic sectors was economic activity^7^. Wang et al. investigated the factors contributing to industrial CO_2_ emissions changes in China from 1994 to 2013, using the LMDI technique. The DA showed that energy intensity of industrial output and energy structure were major determinants of ICE reduction^8^. Zhao et al. analyzed the decoupling effect of economic growth from CO_2_ emissions in China during the period 1992–2012, by employing LMDI. The most important factors affecting decoupling in China were energy intensity and economic activity: the energy intensity of the industrial sector played a key role in promoting the national decoupling state, while the energy emission factor of the agricultural sector made a minor contribution^9^.

Zhang et al. conducted an LMDI DA of the driving factors influencing China's CO_2_ emissions by examining 41 industry subsectors during 2000–2016. Economic growth and energy intensity were crucial factors influencing CO_2_ emissions^10^. Su et al. applied an Index Decomposition Analysis (IDA) framework to compare two groups of countries, G7 and BRICS, regarding the underlying trends in the energy‐related CO_2_ emission during 1990–2015. Energy intensity appeared as the major factor decreasing the CO_2_ emissions in the developed countries (i.e., G7 group)^11^. Shao and Xue adopted the LMDI technique to investigate the evolution of CO_2_ emissions in China in 2000–2016. The results showed that the energy intensity factor was the largest contributor to the reduction of China’s CO_2_ emissions, followed by declining CO_2_ emission intensity^12^. Jiang et al. dealt with the LMDI DA of China’s CO_2_ emissions, investigating influential factors in eight economic regions during 2008–2019. The results indicated that the population size effect was responsible for the increased CO_2_ emissions^13^.

Other scholars have examined the correlation between CO_2_ emissions and specific economic activities (e.g. telecommunications, trade) for China, Brazil and India by means of a nonlinear regression model^14–16^.

Several researchers have investigated the evolution of CO_2_ emissions from the electricity sector. Among the methods for studying the contributing factors, the index decomposition method (IDA) is the one adopted most frequently^17–25^.

Gu et al. studied the CO_2_ emission reduction in China’s electricity sector employing the LMDI technique. They found that the most important factor inducing CO_2_ emission was final electricity consumption^26^. Mousavi et al. employed an LMDI decomposition analysis to examine the driving forces of carbon intensity of electricity generation for Iran. The study indicated that economic activity is the largest driving force of increases in CO_2_ emissions^27^.

Zhang et al. carried out an analysis of electricity consumption in China (1990–2016) using IDA and a decoupling approach. The results showed that electricity consumption exhibited weak decoupling with GDP growth, which indicates that electricity consumption rose with the rise of GDP, while the economic activity effect was the main driving force increasing electricity consumption in China from 1990 to 2016^28^.

Several recent studies have employed decoupling analysis in order to investigate the transition to a less carbon-intensive economy in BRICS. Wang and Jiang used the decoupling index to measure the decoupling states and the driving factors affecting CO_2_ emissions in BRICS. The results denoted that Brazil promotes the decoupling process, in contrast to the performance of the other economies^29^. Ozturk et al. also conducted decoupling and LMDI DA to examine the relationship between CO_2_ emissions and economic growth in Pakistan, India and China. The study determined that India mostly experienced weak decoupling, as well as China^30^. Abam et al. applied LMDI DA and decoupling analysis to study the energy and environmental status of Nigeria’s transport sector. Only the economic structure factor promoted decoupling^31^. Naseem et al. investigated the linkage between economic growth and CO_2_ emissions in BRICS by applying decoupling analysis. The analysis showed that economic expansion and CO_2_ emissions are interrelated in the long run^32^.

There is both research and a policy relevant interest to conduct a comparative study of the energy and climate status in the economies of Brazil, China and India, these being among the power houses of current and forecast growth at the global level. These three BRICS economies have signed the Kyoto Protocol and Paris Agreement, while they are listed as non-Annex I parties of the United Nations Framework Convention on Climate Change (UNFCCC). The main climate targets of these three countries for the study period 1990–2018 are summarized below:

- The Nationally Determined Contribution (NDC) outlined that in the pursuit of low carbon growth, India would reduce the emissions intensity of its GDP by 33–35% from the 2005 level by 2030. Moreover, in 2015, the Indian government announced the aim of achieving 40% of electric power installed capacity from non-fossil fuels by 2030^33^.
- Regarding the Chinese economy, The Revolution Strategy of Energy Production and Consumption (2016–2030) issued in December 2016, committed to reducing carbon intensity in 2030 by 60–65% based on the 2005 level^10^.
- Brazil is committed to reducing greenhouse gas emissions by up to 37% by 2025 and 50% by 2030; 2005 is the reference year^34^.

The objective of the present study is to investigate the influence of driving forces of CO_2_ emissions within the nexus of Economy-Energy-Emissions which is delineated with the findings of Decoupling Analysis. Decoupling Analysis sets the broader relevant picture identifying the macrotrends. The findings of Decomposition Analysis could then interpret them more precisely, and the relative power of individual factors could be estimated more precisely.

The Decomposition Analysis evaluates and ranks the driving forces of CO_2_ emissions, both for energy-related (income, energy intensity, emission factor, energy structure) and power-related emissions (income, energy intensity, emission factor, average household size, number of households). Furthermore, influenced by new contributions to the link between Environment and Society, the study aims to investigate the impact of household dynamics, as household is at the heart of society while the number of households increased disproportionately with population^35–38^.

China, India and Brazil hold a leading regional role in economic and geopolitical terms while are among the drivers of the global growth. They present distinct demographic trends as reflected in the trends of population, household size and number of households. Furthermore, they present different energy status, with Brazil relying relatively more heavily on renewable sources, which makes their comparative analysis interesting. The selection of the period is arbitrary but influenced by two factors: the availability of household data and by the relevant literature that emphasizes the recent period after 2000.

The findings of the analysis could be revealing, however we translate them to policy recommendation with caution. Instead, we suggest future research directions which could support robust policy recommendations together with the findings of the present study.

## Methods

### Decomposition analysis

In the proposed framework of analysis, the time series multiplicative LMDI technique is employed to assess the evolution of the decomposition factors on energy-related CO_2_ and power-related CO_2_ emissions for the three selected economies, during the 2000–2018 period. An intensive report on the time series multiplicative LMDI technique is provided in Refs.^39–42^.

The following variables are defined for each year:

*i*: Fuel type (coal, oil, natural gas, renewable resources)
*E*_*i*_: Energy consumption of fuel type i (Mtoe)
*E*: Total energy consumption (Mtoe)
*C*: Total CO_2_ emissions (MtCO_2_)
*C*_*i*_: CO_2_ emissions from fuel type i (MtCO_2_)
*Y*: GDP (million 2010US$)
*P*: Population (in million people)

The energy-related CO_2_ emissions (C) are given in Eq. (1):

1 $$C = \sum_{i=1}^{4}I {S}_{i} P\,\,Inc\,\, {F}_{i}=\sum_{i=0}^{4}\left(\frac{E}{GDP}\right)\left(\frac{{E}_{i}}{E}\right)P\left(\frac{GDP}{P}\right)\left(\frac{{C}_{i}}{{E}_{i}}\right)$$

where

2 $$\mathrm{Energy \,\,Intensity \,\,}I= \frac{E}{GDP}$$

3 $$\mathrm{Energy\,\, Structure\,\, }{S}_{i}= \frac{{E}_{i}}{E}$$

4 $$\mathrm{Income\,\, }Inc=\frac{GDP}{P}$$

5 $$\mathrm{Emission\,\, factor \,\,}{F}_{i}= \frac{{C}_{i}}{{E}_{i}}$$

6 $$\mathrm{Population \,\,P}$$

The ratio change in CO_2_ emissions levels between 2 years (0 − T) is decomposed to give:

7 $$ D_{{{\text{tot}}}} = D_{{\text{p}}} D_{{{\text{inc}}}} D_{{{\text{int}}}} D_{{\text{f}}} D_{{\text{s}}} = C_{{\text{T}}} /C_{0} $$

where *D*_tot_ is the change of total CO_2_, *D*_p_ is the change of population, *D*_inc_ the change of income, *D*_int_ the change of the energy intensity, *D*_f_ the change of the emission factor and *D*_s_ the change of the energy structure. The formulae for the decomposition factors are presented in the Supplementary File (Equations S1–S5).

**Figure 1:**
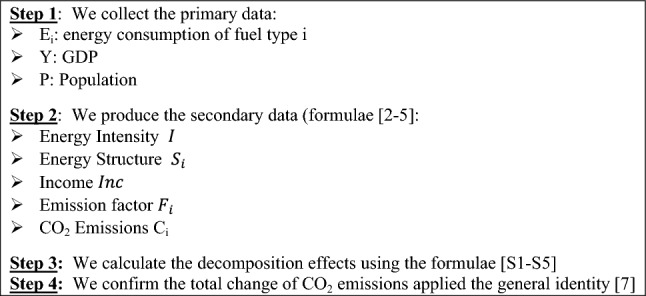
A step by step procedure for the empirical implementation of the Multiplicative LMDI technique.

A step by step procedure for the empirical implementation of the Multiplicative LMDI technique is shown in Fig. 1.

The decomposition factors of power-related CO_2_ emissions (coal, oil and natural gas) for China, India and Brazil are investigated by employing Eq. (8).

8 $$C = \sum_{i=1}^{3}I \,\,Inc \,\,HHN \,\,{AHS \,\,F}_{i}=\sum_{i=0}^{3}\left(\frac{E}{GDP}\right)\Bigg( \frac{GDP }{P} \Bigg)\Bigg( \frac{P }{AHS} \Bigg)AHS \Bigg( \frac{{C}_{i}}{{E}_{i}} \Bigg)$$

where Average household size (number of members) *AHS*,

9 $$\mathrm{Number\,\, of \,\,households }\,\,HHN=\frac{P}{AHS}$$

Similarly, the ratio change in CO_2_ emissions levels between 2 years (0 − T) is decomposed to give:

10 $$ {\text{D}}_{{{\text{tot}}}} = {\text{ D}}_{{{\text{int}}}} {\text{D}}_{{{\text{inc}}}} {\text{D}}_{{{\text{hhn}}}} {\text{D}}_{{{\text{ahs}}}} {\text{D}}_{{\text{f}}} = {\text{ C}}_{{\text{T}}} /{\text{C}}_{0} $$

where D_tot_ is the change of total CO_2_, D_inc_ the change of income, D_f_ the change of the emission factor, D_ahs_ the change of the average household size and D_hhn_ the change of the household number. The relevant formulae for the decomposition factors are given in the Supplementary File (Equations S6–S10).

### Decoupling analysis

With this kind of analysis, we attempt to interpret the link between economic growth and energy-related CO_2_ emissions by means of a decoupling analysis. Energy intensity, as the key index of the link between economy and energy consumption, is often used to assess the energy efficiency of a particular economy, indicating how the economy “converts” energy into monetary output^43^.

The Decoupling Analysis investigates and depicts the macrotrends of the nexus Economy-Energy-Emissions. Energy Intensity and Carbon Intensity are estimated following both traditional (*EI*_*GDP*_ = *E/GDP*) and new indexes (*EI*_*Inc*_ = *E/Income*)^44–46^. Similarly, Emission Intensity is estimated through *CI*_*GDP*_ = *C/GDP* and *CI*_*Inc*_ = *C/Income* ratios^41^. Next, the Decoupling Index (*DI*) for *C/GDP* ratio^47^ is estimated as follows:

11 $$DI_{GDP}=\frac{\Delta (C)}{\Delta (GDP)}=\frac{({C}_{{t}_{1}}-{C}_{{t}_{0}})/{C}_{{t}_{0}}}{({GDP}_{{t}_{1}}-{GDP}_{{t}_{0}})/{GDP}_{{t}_{0}}}$$

The estimation of decoupling of energy-economic growth based on the Energy/Income ratio emerges as an improvement that better approaches the physiology of economic production process, whose specific properties vanish through the use of pure monetary units such as GDP. Remarkably, the indexes DI_Inc_ and CI_Inc_ denote weaker decoupling trends than the DI_GDP_ and CI_GDP_ respectively^41,44–46^.

All these ratios are indexed to a base year (base year = *t*_*0*_ = 100), according to the formulae:

12 $$Indexed\,\, Value\,\, t1 = 100 + \frac{(Value\,\, {t}_{1}-Value \,\,{t}_{0})}{Value \,\,{t}_{0}}$$

We study the decoupling states of India, Brazil and China for the period 2000–2018, based on Tapio’s decoupling model. In order to take into account the impacts of the financial crisis on the link between economy and energy/emission, we focus on changes in the following sub-periods: 2000–2007, 2007–2013 and 2013–2018. These periods correspond to: before the Great Recession (2000–2007); the Great Recession (2007–2013); and after the Great Recession (2013–2018). This kind of analysis has been proposed by various studies^41,48^.

## Data analysis

Regarding India, China and Brazil, the data on Energy Consumption are drawn from the International Energy Agency^2^. To estimate CO_2_ emissions from primary fuel consumption we adopted the OECD emission coefficients^49^, as shown in Table S1.

All data on GDP, Population and Income are retrieved from World Bank Open Data^50^. The key variables of our study are presented for the period 2000–2018 in Table S2.

In 2010 the Chinese economy surpassed the USA economy in the world energy rankings^51^. During the 2000–2018 time period, China and India increased their energy consumption remarkably by 163.4% and 92.3% respectively, while Brazil’s energy consumption grew by 46.3%.

As shown in Table S2, China increased CO_2_ emission by 171.4% for the period 2000–2018, India by 153.3%, and Brazil by 28%. China’s GDP sky-rocketed from 2232 to 10,797 billion dollars (384% growth) during the study period. Brazil’s economy followed with a significant growth of 225.4%. India’s GDP also grew rapidly (50.1%).

Table S3 depicts the EI_GDP_, EI_Inc_, CI_GDP_ and CI_Inc_ indexes for India, Brazil and China. We also calculate the percentage changes in the relevant indexes from 2000 to 2018. Table S3 indicates that the Chinese economy had a similar reduction of each index during the study period. For India, the EI_GDP_ reduction was almost twice the reduction of CI_GDP_, while the reduction of Brazil’s EI_GDP_ was only marginal (− 2.5%).

Figure S1.a,b. presents the Emission Factor (F) and Energy Intensity (EI) in the electricity and heat sectors respectively for the three economies. All values are indexed to 2000 as base year (2000 = 1).

Brazil’s emission factor from electricity and heat generation presented significant fluctuation in the period 2006–2010, given an unstable energy policy^52^. During 2011–2014 the emission factor effect sky-rocketed (+ 109%) due to severe political and economic crisis^7,11^. Eventually, in the later years of the study period (2015–2018), the Brazilian economy followed a sustainable path (− 70%). China’s emission factor from electricity and heat generation declined by 37% in the study period, while India’s emission factor also declined, by 21%.

China has been the leading producer and consumer of electricity since 2011. Given its over-dependence on coal, electricity and heat generation accounts for more than half of the total CO_2_ emissions in China and 15% of the total CO_2_ emissions in the world^53,54^ (Fig. S1b).

Figure S2 presents electricity and heat generation percentages by fuel type for (a) China, (b) Brazil and (c) India in 2000 and 2018. Brazil largely relies on hydropower for electricity and heat generation; in 2020, hydropower supplied 66% of its electricity and heat demand. Brazil's hydroelectric potential mainly lies in the Amazon River^55,56^. For the economies of China (Fig. S2.a) and India (Fig. S2.c) electricity and heat generation from coal takes the lion’s share of total production, a state that implies that these economies need to take on the challenge of having more renewables-oriented electricity and heat generation.

As presented in Fig. S3. CO_2_ emissions from electricity and heat producers for India and China follow similar upward trends. Brazil’s trendline fluctuated through the study period and declined from 2014.

Figure S4 demonstrates CO_2_ emissions from electricity and heat by energy source for each economy.

For the case of India (Fig. S4.a) coal increased by 177% and oil decreased by − 54%. These significant changes are possibly linked to the liberalization of gasoline (2010) and diesel (2013) prices in India^57^.

Power-related CO_2_ emissions from gas increased in Brazil and China by 11% and 18.5% respectively. The coal and oil levels followed similar, almost stationary trends in Brazil (Fig. S4.b) and China (Fig. S4.c). More specifically, the wake of Brazil’s offshore pre-salt hydrocarbon discoveries in 2007 and 2008 present the prospect of Brazil becoming a Liquefied Natural Gas exporter^58^. Regarding China, the rising levels of urbanization has led to the growth of energy demand. Simultaneously, the advanced expectations of response to environmental pressures conclude in greater attention to use of gas, which drives policy reform^59^.

Data for Average Household Size (AHS) are retrieved from the Institute of Management Research—Radboud University^60^ and presented in Fig. S5. Brazil AHS data after 2013 are calculated based on the average annual rate of increase for the period 2000–2013 (− 1.3%). The AHS data for Brazil and India decreased by 21% and by 18% respectively during the study period but increased for China by 11.6%. This fact probably indicates de-growth in living standards in China due to heavy industrialization in the contemporary years.

The data sources for each economy are listed in Table S4.

## Results and discussion

**Figure 2:**
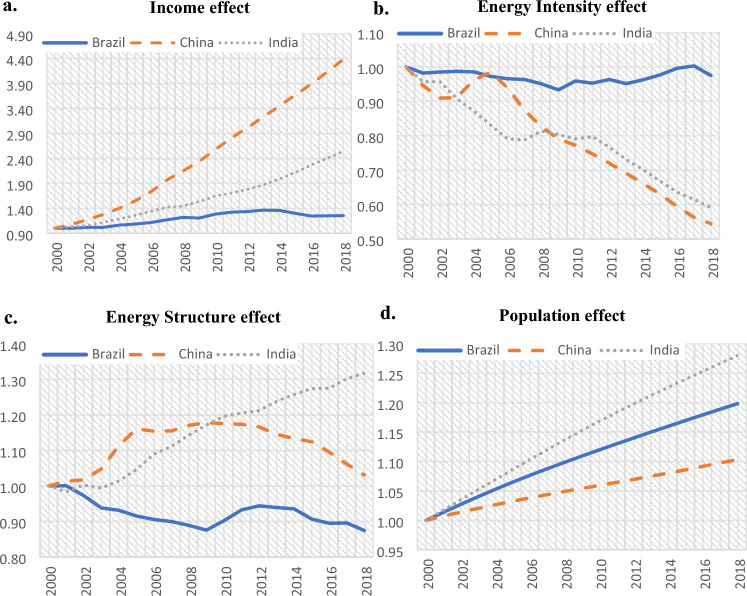
(**a**) Change of energy related CO_2_ emissions due to the Income Effect. (**b**) Change of energy related CO_2_ emissions due to the Energy Intensity Effect. (**c**) Change of energy related CO_2_ emissions due to the Energy Structure Effect. (**d**) Change of energy related CO_2_ emissions due to the Population Effect.

We apply the Multiplicative LMDI technique to energy-related CO_2_ emissions to explore and rank the selected decomposition effects as systematically represented in Fig. 2a–d for the period 2000–2018.

**Figure 3:**
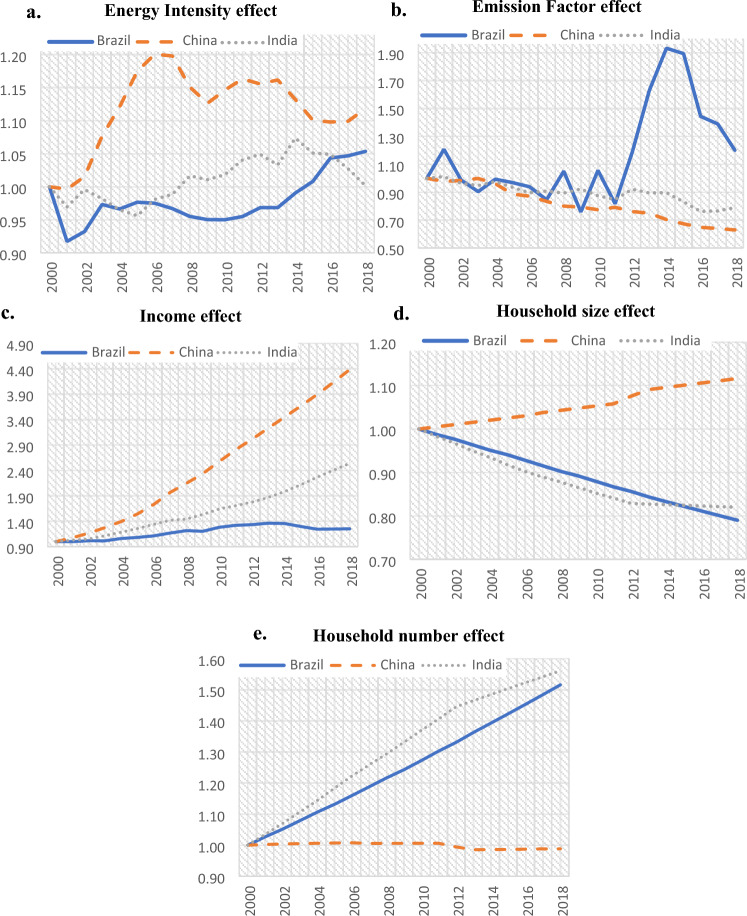
(**a**) Change of power related CO_2_ emissions due to the Energy Intensity Effect. (**b**) Change of power related CO_2_ emissions due to the Emission Factor Effect). (**c**) Change of power related CO_2_ emissions due to the Income Effect. (**d**) Change of power related CO_2_ emissions due to the Household size Effect. (**e**) Change of power related CO_2_ emissions due to the Household number Effect.

Multiplicative LMDI estimates for power-related CO_2_ emissions are shown in Fig. 3 and Table 1 ranks selected decomposition effects for the period 2000–2018.

**Table 1:** Rank of the overall contribution (2000–2018) of LMDI decomposition effects*.*

	China	India	Brazil
1st	*D*_*inc*_ (+ 338%)	*D*_*inc*_ (+ 154%)	*D*_*hhn*_ (+ 52%)
2nd	*D*_*ahs*_ (+ 12%)	*D*_*hhn*_ (+ 56%)	*D*_*inc*_ (+ 25%)
3rd	*D*_*int*_ (+ 12%)	*D*_*int*_ (infinitesimal)	*D*_*f*_ (+ 20%)
4th	*D*_*hhn*_ (infinitesimal)	*D*_*ahs*_ (− 18%)	*D*_*int*_ (+ 5%)
5th	*D*_*f*_ (− 37%)	*D*_*f*_ (− 21%)	*D*_*ahs*_ (− 11%)

Tables 2, 3 and 4 present the decoupling states for India, Brazil and China, respectively, for the period 2000–2018, based on Tapio’s decoupling model.

**Table 2:** Decoupling analysis of energy-related CO_2_ emissions for 2000–2018 in India.

Economy	DI_GDP_	Decoupling state	DI_Inc_	Decoupling state
2000–2007	0.66	Weak decoupling	1.68	Strong decoupling
2007–2013	1.11	Expansive coupling	26.53	Expansive decoupling
2013–2018	0.54	Weak decoupling	5.85	Expansive decoupling

**Table 3:** Decoupling analysis of energy-related CO_2_ emissions for 2000–2018 in Brazil.

Time period	DI_GDP_	Decoupling state	DI_Inc_	Decoupling state
2000–2007	0.38	Weak decoupling	2.58	Expansive decoupling
2007–2013	1.16	Expansive coupling	36.24	Expansive decoupling
2013–2018	2.04	Expansive decoupling	− 1.18	Strong decoupling

**Table 4:** Decoupling analysis of energy-related CO_2_ emissions for 2000–2018 in China.

Time period	DI_GDP_	Decoupling state	DI_Inc_	Decoupling state
2000–2007	1.02	Expansive coupling	7.11	Expansive decoupling
2007–2013	0.47	Weak decoupling	5.35	Expansive decoupling
2013–2018	− 0.03	Strong decoupling	− 0.23	Strong decoupling

### The case of China

Regarding the DA of energy-related CO_2_ emissions, the income effect indicates a remarkably strong positive contribution to the increase of energy-related CO_2_ emissions in China (+ 338%), while the energy intensity effect had a powerful negative contribution (-54%). The energy structure effect was positive, reaching its maximum in 2008 (+ 18%, 2000–2008), but the overall contribution was weak (+ 3%). The Chinese population effect contributed 10%.

In the DA of the power-related emissions, the energy intensity effect had a positive contribution of 12%, and the emission factor effect a negative contribution (− 37%). The socioeconomic factors also presented interesting results; the household size effect in China made a positive contribution (+ 12%), whereas the number of households had an almost negligible impact (− 1%). Remarkably, China’s household size has been increasing while the number of households remained relatively stable, as the result of an increasing population facilitated by an increasing household size.

China presents progress according with the goal of emissions reduction marking a strong decoupling in the period 2000–2018 by − 44% in the emissions intensity of one unit of GDP (Table S3c), following a similar trend in the respective energy intensity.

### The case of India

The income effect in India indicates a strong positive contribution to the increase of energy-related CO_2_ emissions (+ 154%), while the energy intensity effect made an overall negative contribution to the evolution of CO_2_ emissions (− 41%). India’s energy structure effect had a strong positive contribution (+ 32%) in the increase of CO_2_ emissions. The population effect contributed 28%.

Regarding the DA of power-related CO_2_ emissions, the trend of energy intensity for the Indian economy had a trivial effect, while the contribution of the emission factor effect was negative (− 21%). Household size made a negative contribution (-18%) and the number of households had a strong positive effect (56%). Increasing household numbers influence the use of power and hence increase the corresponding emissions; this impact overcomes the negative impact induced by the decreasing size of households with lower power use per household.

In the Decoupling Analysis, the Indian economy culminates in a weak/expansive decoupling status, marking a decrease by 22% in the emission intensity of one unit of GDP, substantially lower than the respective decrease of the energy intensity (− 41%). Remarkably, the DI_inc_ index (expansive decoupling) underlines the attempt of India’s economy to move towards a strong decoupling.

### The case of Brazil

There was an overall decrease of CO_2_ emissions of 28% in the period 2000–2018 with the energy structure effect leading this trend with a negative contribution of − 13%. Evidently, the energy structure effect has been induced by the relatively high share of renewables (27%). This is a distinctive feature of Brazil’s energy structure. The income effect indicates a positive contribution to the increase of energy-related CO_2_ emissions (+ 25%). The Brazilian energy intensity effect presents a weak negative contribution (-3%), possibly due to the growth of the Brazilian renewables by 81% in the same period^2^. Indeed, the small influence of EI could be attributed to the significant impact of an energy structure dominated by the high and still increasing use of renewables. The population effect contributed 20% and the income effect had a positive contribution (+ 25%).

Regarding electricity DA, the energy intensity effect induced a small increase of 5%, and the emission factor effect presented a positive contribution (20%) with several fluctuations. The household size effect contribution was negative (− 11%) during the study period, while the number of households made a strong positive contribution (+ 52%), a result with similar interpretation as for India.

Regarding Decoupling Analysis, the Brazilian economy achieved the reduction among these economies in its emission intensity (− 15%) which is however higher than the infinitesimal reduction of the respective energy intensity (− 2.5%).

### Comparison with other studies

In the energy and environmental literature there are several studies focusing on driving factors of energy-related and power-related CO_2_ emissions on a comparative basis for different economies. The crucial findings of our research coincide with several studies; Zhao et al. concludes that the key driver for the increase of CO_2_ emissions was the economic activity, while energy intensity was the biggest contributor to declining CO_2_ emissions in India and China^9^. Attari and Attaria, Das and Paul, Yeo et al. and Rüstemoglu and Andrés reported similar findings^4–7^.

Regarding power-related analysis, our results are similar to the work of Mousavi et al. and Zhang et al.; energy intensity and emission factor effects are presented as the main driving forces responsible for the decrease and increase of energy-related CO_2_ emissions, respectively^27,28^. The decoupling analysis of Wang and Jiang indicates that Brazil promotes the decoupling process, in accordance with our findings^29^.

## Conclusions

This study attempts a DA and decoupling analysis of the energy and power related CO_2_ emissions, for the economies of China, India and Brazil during 2000–2018. These three economies have a large environmental impact, being rapidly emerging economies with great influence on the regional and global geopolitical status in the coming years. The analysis highlights the driving factors which affect the changes in CO_2_ emissions in the selected BRICS countries for two major reasons:

- the significant contribution of BRICS to the total amount of CO_2_ emissions (energy-related & power-related) at the global level.
- BRICS countries do not have legally binding emissions reductions targets (Annex-I countries).

Regarding the influence of the different factors, the following findings are dominant. The raw decoupling estimates (Table S3), delineating the nexus of Economy-Energy-Emissions, suggest that the emission intensity is clearly influenced by the energy intensity, as they follow similar trends. There is a substantial difference between the Energy/Emission Intensity of one unit of GDP (EI_GDP_ & CI_GDP_) and Energy/Emission Intensity of one unit of Income (EI_Inc_ & CI_Inc_). The Energy Intensity of one unit of Income has been increasing for the period at hand, inducing a more intensive increase of the respective Emission Intensity, with the exception of Brazil which presents a breakdown trend in its Income Emission Intensity with − 2.1% reduction. In contrast to the Energy/Emissions Intensity of Income, the Energy/Emission Intensity of GDP has been decreasing with China having the greatest reduction.

The estimates of the Decoupling Index (Tables 2, 3, 4) indicated that the Chinese economy presents strong decoupling during the period 2013–2018, being the only country moving towards the goals of CO_2_ mitigation. Despite their efforts, Indian and Brazilian economies have a weak decoupling status (the growth rate of CO_2_ is less than that of the economy) or expansive decoupling status (CO_2_ emissions grow faster than the GDP) within the last 11 years.

The energy-related CO_2_ emissions are influenced positively by increasing income and population in all countries. The increasing, income-based, economic well-being for a growing population induced CO_2_ emissions. On the contrary, the reduced energy intensity, energy required for one unit of GDP, influences CO_2_ emissions negatively. The exception in the case of Brazil is because of the high effect of the energy structure reflecting the high and still increasing impact of renewables. Energy structure has an important negative impact in China, while the energy structure in India contributes positively.

The power-related emissions are influenced negatively by the emission factor in China and India; in contrast this factor influences positively the Brazilian power-related emissions. Remarkably, the energy intensity influences positively China and has marginal impacts on India and Brazil. The power-related emissions are strongly linked with the power use of households, which are among the major end-users. Household size has a significant positive impact in China where household size increased and a negative impact in Brazil and India where household size decreased. The increasing number of households results in an intensively positive effect in Brazil and India; China’s effect is not relevant as its number of households is fairly stable.

The impact of households indicates the significance of the coupled human, nature and economic systems. Energy and climate policies need to take into account social conditions and evolutions. Indicatively, the promotion of energy saving practices in the domestic sector is of great importance for policy makers. Improvements in household energy efficiency could be achieved with the implementation of several actions such as the use of small renewable sources to increase the share of renewable energy use and a long-term renovation strategy of buildings (similar to programs already existing in the European Union).

The findings of the study further indicate that emissions are inevitably defined by the use of energy and its structure. Several additional factors influence the emissions trends. Increasing well-being of an increasing population leads to a higher level of emissions. However, as the case of Brazil indicates, the ultimate outcome will be mediated by the role of the energy mix. These findings are important for influencing energy policies in rapidly growing economies in Asia and South America. Nevertheless, before concrete policy recommendations can be sharply drawn, scientific research should endeavor to investigate the driving forces in those economic sectors with high climate impacts, such as transport, agriculture etc. The armament of the scientific analysis can be enriched by applying econometric methods, which can investigate causal relationships among variables.

The analysis could be extended including more socio-demographic factors (e.g. education, dwelling type), psychological factors (e.g., knowledge, values, attitudes, motivations, intentions, social norms) and external contextual and situational factors (e.g. socio-cultural, political, legal, institutional forces) in an integrated econometric model. The incorporation of these factors would offer new insights into the relationships among Energy, Society and Environment.

### Supplementary Information

Supplementary Information.

## Data Availability

The datasets used and/or analyzed during the current study are available from the corresponding author on reasonable request.
