# Supplementary material for: Social and economic driving forces of recent CO2 emissions in three major BRICS economies
Source: Sci Rep. 2024 Apr 5;14:8047. doi: 10.1038/s41598-024-58827-9 (PMC10997659; doi:10.1038/s41598-024-58827-9)
Supplement: Supplementary file 1 — Supplementary Information. [file 41598_2024_58827_MOESM1_ESM.pdf]

Social and economic driving forces of recent CO_2 emissions in three major BRICS economies.

Eleni Koilakou[a*], Emmanouil Hatzigeorgiou[a,c], Kostas Bithas[a,b]

a. Institute of Urban Environment & Human Resources, Department of Economic & Regional Development, Panteion University, 29 Aristotelous Street, GR-17671, Kallithea, Athens, Greece

b. Center for Systems Integration & Sustainability, Michigan State University, Manly Miles Building, 1405 South Harrison Road East Lansing, MI 48823-5243, USA

c. Energy Management Laboratory, Department of Environment, University of the Aegean, University Hill, 81100, Lesvos, Greece

*Corresponding author at: Institute of Urban Environment & Human Resources, Department of Economic & Regional Development, Panteion University, 29 Aristotelous Street, GR-17671, Kallithea, Athens, Greece.
E-mail addres: elenikoilakou@gmail.com .

Supplementary Information File

A. Supplementary Figures

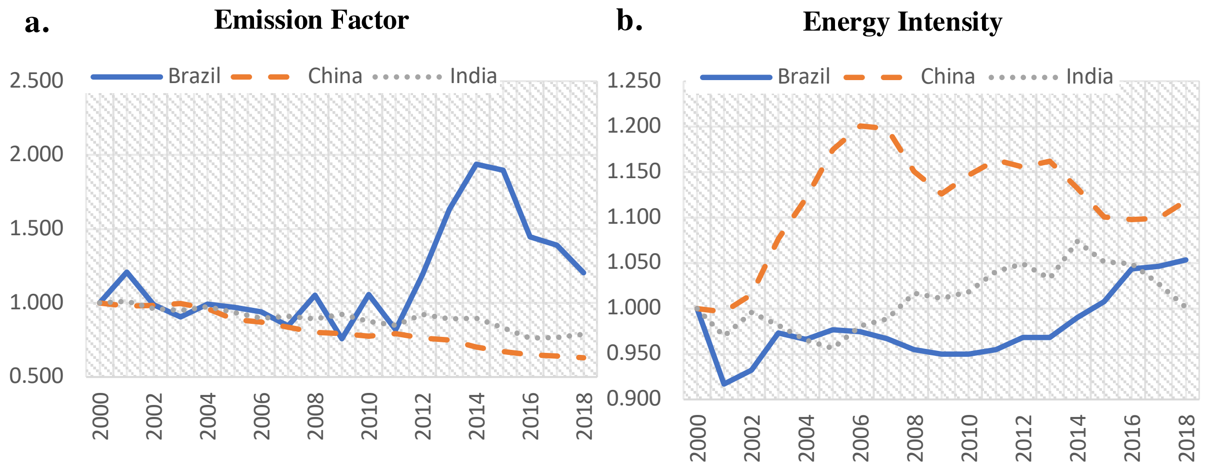

Fig.S1. a. Emission factor in electricity and heat sector for India, China & Brazil from 2000 to 2018, indexed (2000=1). **b.** Energy Intensity in electricity and heat sector for India, China & Brazil from 2000 to 2018, indexed (2000=1).

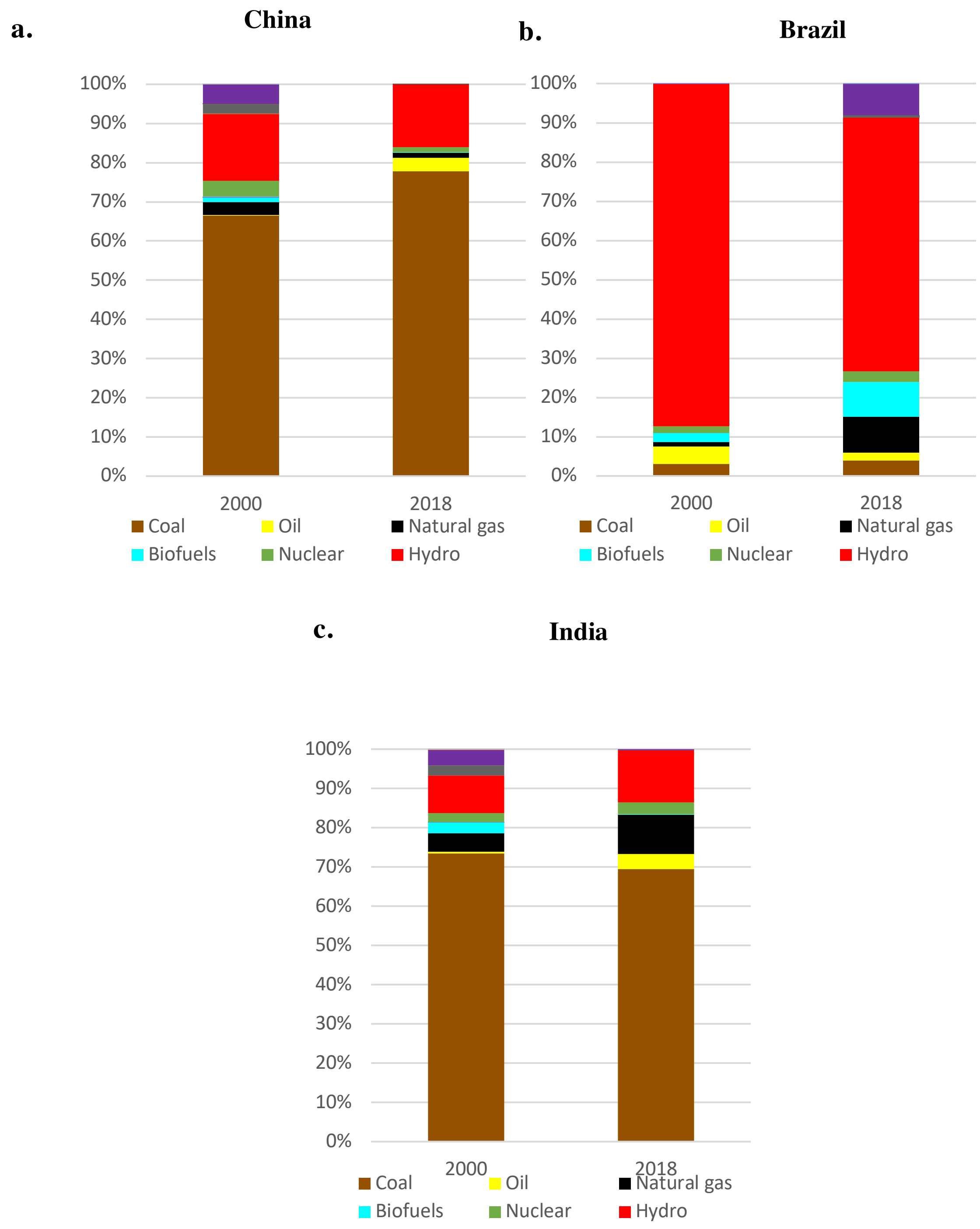

Fig.S2. Electricity and heat generation percentages by fuel for (a) China, (b)Brazil and (c)India in 2000 and 2018.

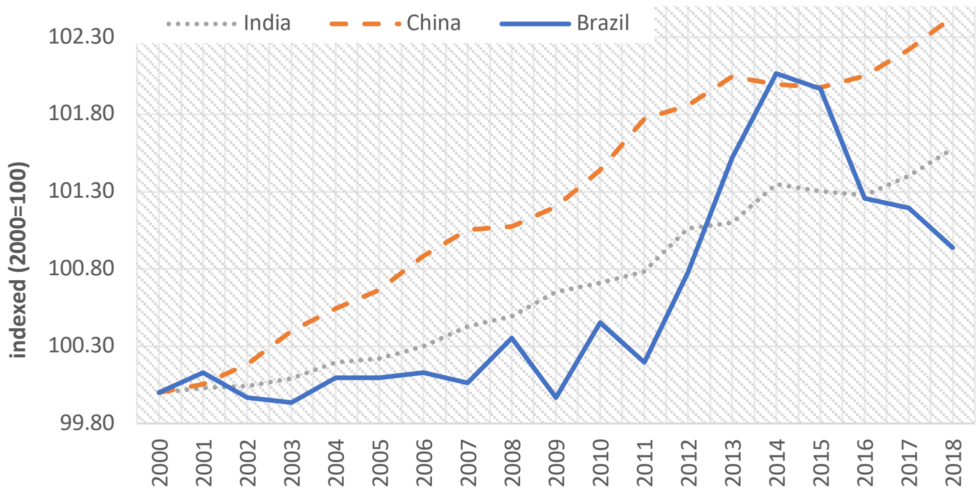

Fig. S3. CO_2 emissions from electricity and heat producers for India, China & Brazil from 2000 to 2018, indexed (2000=100).

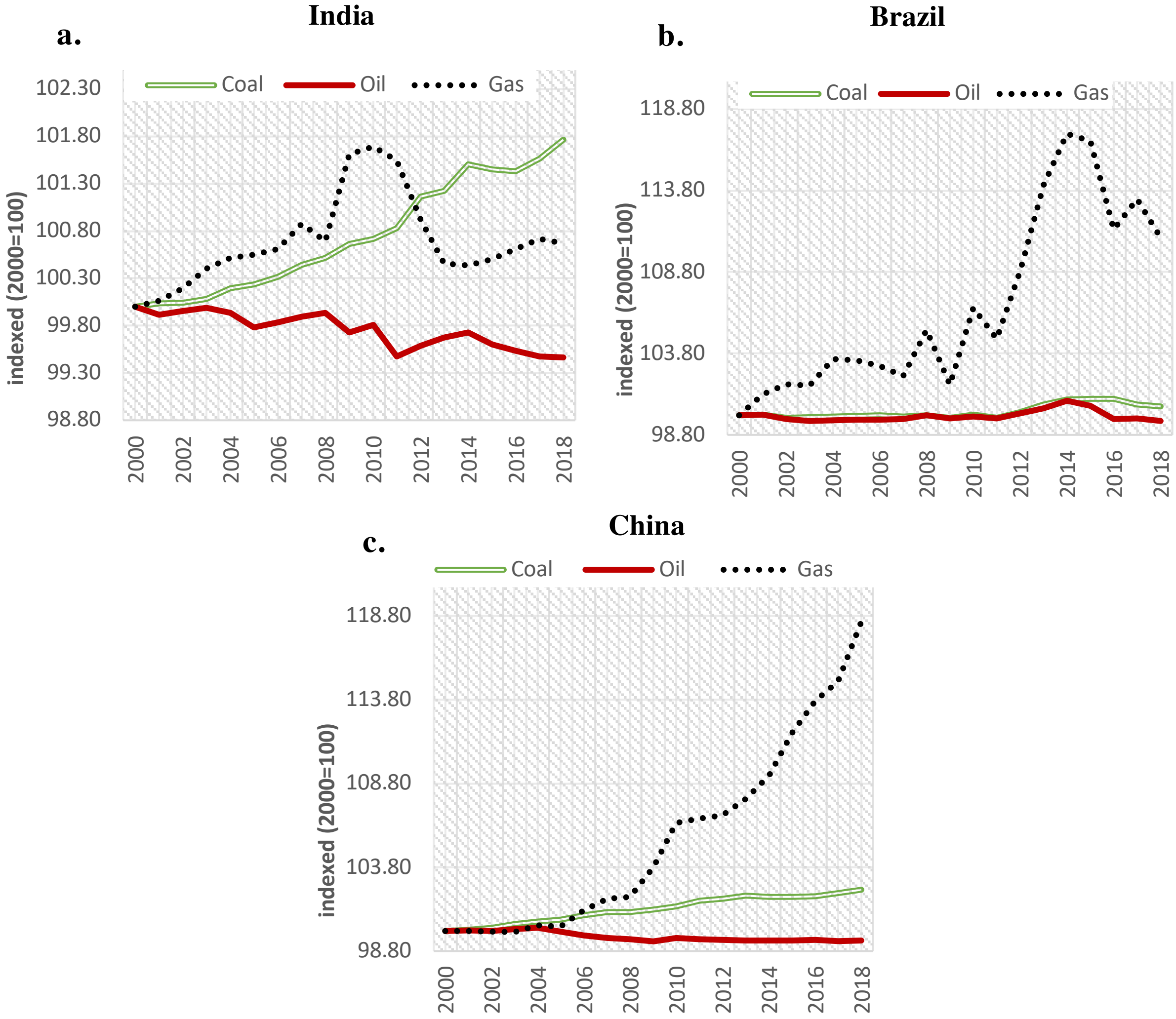

Fig. S4. a. CO_2 emissions from electricity and heat by energy source, India 2000-2018, indexed (2000=100) **b.** CO_2 emissions from electricity and heat by energy source, Brazil 2000-2018, indexed (2000=100). **c.** CO_2 emissions from electricity and heat by energy source, People's Republic of China 2000-2018, indexed (2000=100).

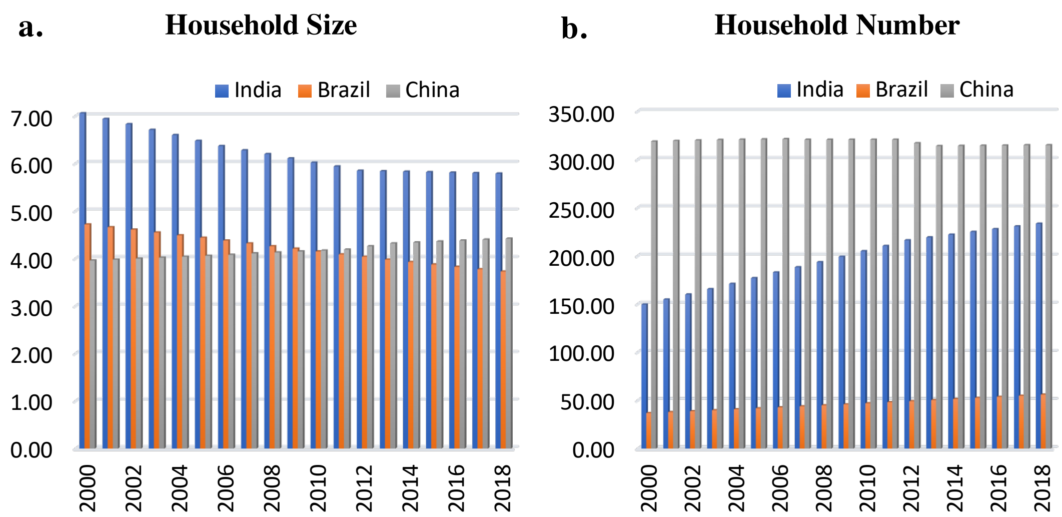

Fig. S5. a. Average Household Size (AHS) for India, Brazil & China from 2000 to 2018
b. Household Number (HHN) for India, Brazil & China from 2000 to 2018

B. Supplementary Tables

Fuel	Coefficient
Coal	3.99 tCO_2/toe
Oil	3.07 tCO_2/toe
Natural Gas	2.35 tCO_2/toe
Renewable Resources	0.00 tCO_2/toe

Table S1. CO_2 emission coefficients

a. *Key variables - China*	**2000**	**2018**	**% Change**
**Energy Consumption** (*Mtoe*)	781,2	2.057,7	**163,4**
**CO_2 emissions** (*$MtCO_2$*)	1.671,0	4.535,7	**171,4**
**GDP** (*million 2010 US$)*	2.232.146,3	10.797.222,2	**384**
**Population** (*million people*)	1.262,6	1.392,7	**10,3**
**Income** (*2010 US$*)	1.767,9	7.752,7	**338,5**
**Household number** *(in millions)*	318,85	315,10	**-1,2**

b. *Key variables - Brazil*	**2000**	**2018**	**% Change**
**Energy Consumption** (*Mtoe*)	153,5	224,6	**46,3**
**CO_2 emissions** (*$MtCO_2$*)	280,3	358,7	**28**
**GDP** (*million 2010 US$)*	1.538.706,0	2.309.659,4	**50,1**
**Population** (*million people*)	174,8	209,5	**19,9**
**Income** (*2010 US$*)	8.802,7	11.024,6	**25,25**
**Household number** *(in millions)*	37,03	56,20	**51,8**

c. *Key variables - India*	**2000**	**2018**	**% change**
**Energy Consumption** (*Mtoe*)	315,5	606,6	**92.3**
**CO_2 emissions** (*$MtCO_2$*)	449,8	1.139,2	**153,3**
**GDP** (*million 2010 US$)*	873.357,4	2.841.579,5	**225,4**
**Population** (*million people*)	1.056,6	1.352,6	**28**
**Income** (*2010 US$*)	826,6	2.100,8	**154,1**
**Household number** *(in millions)*	149,66	233,61	**56,1**

Table S2. Key variables for China, Brazil and India economies.

a.

2000

**Economy**	EI_{GDP}	CI_{GDP}	EI_{Inc}	CI_{Inc}
Brazil	0,000100	0,000182	0,003901	0,007126
India	0,000361	0,000515	0,008019	0,011433
China	0,000350	0,000749	0,017620	0,037691

b.

2018

**Economy**	EI_{GDP}	CI_{GDP}	EI_{Inc}	CI_{Inc}
Brazil	0,000097	0,000155	0,004371	0,006979
India	0,000213	0,000401	0,011803	0,022166
China	0,000191	0,000420	0,036185	0,079764

c.

**Economy**	EI_{GDP}	CI_{GDP}	EI_{Inc}	CI_{Inc}
Brazil	-2,50%	-14,76%	12,02%	-2,06%
India	-40,90%	-22,15%	47,18%	93,88%
China	-45,55%	-43,88%	105,36%	111,63%

Table S3. Carbon intensity and energy intensity comparison for the economies of Brazil, India and China for the years a) 2000, b) 2018 and c) percentage change (%) for the period 2000-2018.

Variables	*Economies*
Energy Consumption	International Energy Agency
GDP, Population and Income	World Bank Open Data
CO_2 emissions from primary fuel consumption	OECD emission coefficients
Electricity and heat generation by fuel	International Energy Agency
Average Household Size	Institute of Management Research - Radboud University

Table S4. The list of data sources of key variables for China, Brazil and India economies.

C. Supplementary Equations

i.

$$D_{\mathrm{p}} = \exp\left(\sum_{i=1}^{4} \frac{(C_{i,T}-C_{i,0})/(\ln C_{i,T}-\ln C_{i,0})}{(C_T-C_0)/(\ln C_T-\ln C_0)} \ln\left(\frac{P_T}{P_0}\right)\right) \quad \text{(S1)}$$

$$D_{\mathrm{inc}} = \exp\left(\sum_{i=1}^{4} \frac{(C_{i,T}-C_{i,0})/(\ln C_{i,T}-\ln C_{i,0})}{(C_T-C_0)/(\ln C_T-\ln C_0)} \ln\left(\frac{Inc_T}{Inc_0}\right)\right) \quad \text{(S2)}$$

$$D_{\mathrm{int}} = \exp\left(\sum_{i=1}^{4} \frac{(C_{i,T}-C_{i,0})/(\ln C_{i,T}-\ln C_{i,0})}{(C_T-C_0)/(\ln C_T-\ln C_0)} \ln\left(\frac{I_T}{I_0}\right)\right) \quad \text{(S3)}$$

$$D_{\mathrm{f}} = \exp\left(\sum_{i=1}^{4} \frac{(C_{i,T}-C_{i,0})/(\ln C_{i,T}-\ln C_{i,0})}{(C_T-C_0)/(\ln C_T-\ln C_0)} \ln\left(\frac{F_{i,T}}{F_{i,0}}\right)\right) \quad \text{(S4)}$$

$$D_{\mathrm{s}} = \exp\left(\sum_{i=1}^{4} \frac{(C_{i,T}-C_{i,0})/(\ln C_{i,T}-\ln C_{i,0})}{(C_T-C_0)/(\ln C_T-\ln C_0)} \ln\left(\frac{S_{i,T}}{S_{i,0}}\right)\right) \quad \text{(S5)}$$

ii.

$$D_{\mathrm{int}} = \exp\left(\sum_{i=1}^{3} \frac{(C_{i,T}-C_{i,0})/(\ln C_{i,T}-\ln C_{i,0})}{(C_T-C_0)/(\ln C_T-\ln C_0)} \ln\left(\frac{I_T}{I_0}\right)\right) \quad \text{(S6)}$$

$$D_{\mathrm{inc}} = \exp\left(\sum_{i=1}^{3} \frac{(C_{i,T}-C_{i,0})/(\ln C_{i,T}-\ln C_{i,0})}{(C_T-C_0)/(\ln C_T-\ln C_0)} \ln\left(\frac{Inc_T}{Inc_0}\right)\right) \quad \text{(S7)}$$

$$D_{\mathrm{ahs}} = \exp\left(\sum_{i=1}^{3} \frac{(C_{i,T}-C_{i,0})/(\ln C_{i,T}-\ln C_{i,0})}{(C_T-C_0)/(\ln C_T-\ln C_0)} \ln\left(\frac{AHS_T}{AHS_0}\right)\right) \quad \text{(S8)}$$

$$D_{\mathrm{hhn}} = \exp\left(\sum_{i=1}^{3} \frac{(C_{i,T}-C_{i,0})/(\ln C_{i,T}-\ln C_{i,0})}{(C_T-C_0)/(\ln C_T-\ln C_0)} \ln\left(\frac{AHN_T}{AHN_0}\right)\right) \quad \text{(S9)}$$

$$D_{\mathrm{f}} = \exp\left(\sum_{i=1}^{3} \frac{(C_{i,T}-C_{i,0})/(\ln C_{i,T}-\ln C_{i,0})}{(C_T-C_0)/(\ln C_T-\ln C_0)} \ln\left(\frac{F_{i,T}}{F_{i,0}}\right)\right) \quad \text{(S10)}$$

References

Bureau of Economic Analysis (BEA) (2020) BEA Data. https://www.bea.gov/data/gdp
Intergovernmental Panel on Climate Change (IPCC) (2006) IPCC guidelines for national greenhouse gas inventories. Intergovernmental panel on climate change. London.
International Energy Agency (IEA) (2020a) Data & Statistics. https://www.iea.org/data-and-statistics
The World Bank Group (2020) World Bank Open Data. https://data.worldbank.org
